# Giant Band Gap Narrowing under Hydrostatic Pressure in (4FP)_2_SnI_4_ Halide Perovskite

**DOI:** 10.1021/acs.jpclett.5c00903

**Published:** 2025-06-16

**Authors:** Rafał Bartoszewicz, Jakub Ziembicki, Ewelina Zdanowicz, Artur P. Herman, Jarosław Serafińczuk, Jesús Sánchez-Diaz, Samrat Das Adhikari, Iván Mora-Seró, Robert Kudrawiec

**Affiliations:** † Department of Semiconductor Materials Engineering, 49567 Wrocław University of Science and Technology, WybrzeŻe Wyspiańskiego 27, 50-370 Wrocław, Poland; ‡ Department of Nanometrology, 49567 Wrocław University of Science and Technology, Janiszewskiego 11/17, 50-372 Wrocław, Poland; ¶ Institute of Advanced Materials (INAM), 16748 Universitat Jaume I. Av. de Vicent Sos Baynat, Castellón de la Plana, 12006 Spain; § Institute of Physical Chemistry, Polish Academy of Sciences, Warsaw 01-224, Poland

## Abstract

One of the most intriguing properties of hybrid halide perovskites is their structural softness. The interplay between organic and inorganic sublattices leads to a multitude of physical phenomena that are not observed in conventional semiconductors such as III–V materials. One among these is the exceptional band gap tunability under hydrostatic pressure. In this context, tin-based perovskites exhibit a more pronounced effect than their lead-based counterparts. Here, we report on one of the largest band gap tunabilities in this material family. The studied material, layered tin-based (4FP)_2_SnI_4_ perovskite, exhibits an exceptionally strong pressure sensitivity, with a band gap shift of up to −160 meV/GPa at room temperature. Combined experimental and computational studies reveal that its band gap dependence on pressure remains linear in the 0–5 GPa range, making (4FP)_2_SnI_4_ highly attractive for pressure-sensor applications.

## Graphical abstract

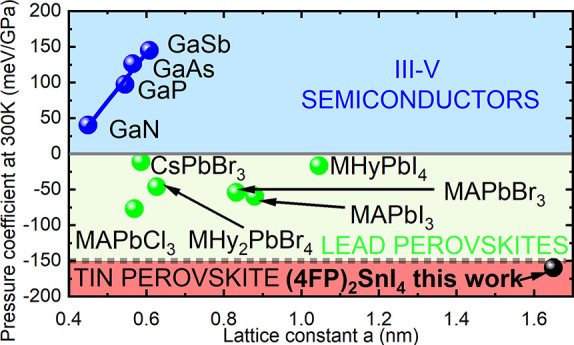

Layered or so-called “two-dimensional” (2D) lead halide perovskites have received considerable attention in recent years due to their high exciton binding energy, high photoluminescence quantum yield, and versatile exciton radiative recombination processes, which are desirable for micro/nanolasers and high-efficiency light-emitting diodes (LED).^1–14^ Nevertheless, the utilization of Pb-based perovskites is constrained by the toxicity of the heavy metal component, which restricts their commercial use in optoelectronic devices. In order to optimize the utility of perovskites, researchers have attempted to substitute Pb with less toxic elements, such as Sn.^15,16^ Sn-based perovskites demonstrate similar or superior electronic and optical properties compared to Pb-based perovskites, such as higher charge carrier mobilities, long-lived hot carriers, low-cost synthesis, and environmental inertness for use in commercial applications.^17–24^ Despite these facts, the main factor that limits the use of Sn-based perovskites for mass-produced devices is their poor structural stability due to the rapid oxidation of the Sn^2+^ state to the Sn^4+^ state under ambient conditions, as well as the abundance of Sn vacancy defects.^25^ One of the representatives of Sn-based halide perovskites is 4-fluorophenethylammonium tin iodide ((4FP)_2_SnI_4_), which is a perovskite suitable for applications such as micro/nanolasers, LEDs, or another applications.^12,26^ To enhance the application prospects of (4FP)_2_SnI_4_, it is necessary to explore its optical properties, especially its response to hydrostatic pressure. Due to the high flexibility of the crystal structure of perovskites, this approach seems to be an appropriate way to modulate the lattice in a controllable manner to tailor their optoelectronic properties, such as the band gap and the intensity of photoluminescence (PL).^27,28^ So far, PL and absorption measurements under hydrostatic pressure have been reported for numerous 2D perovskites.^29–36^ However, such studies are rare for tin-based perovskites, and no reports exist for (4FP)_2_SnI_4_, particularly regarding temperature-dependent experiments.

In this Letter, we report on the PL of near-band gap emission (NBE) and absorption edge studies for (4FP)_2_SnI_4_ perovskite under hydrostatic pressure to determine the narrowing of the band gap and compare it with other semiconductor compounds, particularly Pb-based halide perovskites. We have observed a giant pressure coefficient of −160 meV/GPa for (4FP)_2_SnI_4_, which leads to one of the highest band gap tunability potential above halide perovkites and conventional semiconductors (see graphic in the Abstract).^34,37–48^ Moreover, unlike in conventional semiconductors, the band gap pressure coefficient significantly changes with temperature.

The synthesis of (4FP)_2_SnI_4_ was carried out using the developed methodology, as detailed in the Supporting Information (SI). Specifically, the microcrystals were synthesized by carefully controlling the amount of hydroiodic acid to prevent an excess of iodide and water, which could otherwise compromise the stability of the material. Acetic acid was employed as the solvent, playing a crucial role in stabilizing the perovskite. It was observed that acetic acid helped maintain structural stability by coordinating with surface iodide and tin atoms,^12,56^ thereby reducing the moisture exposure to the material. By following this modified approach, the stability of (4FP)_2_SnI_4_ was significantly enhanced and has been studied in a previous report.^12^ After successful synthesis and fundamental optical characterization at room temperature, we measured XRD patterns as a function of the pressure. Figure 1a displays the XRD patterns of (4FP)_2_SnI_4_ under pressure up to 5.19 GPa at 300 K. All visible diffraction peaks are well indexed assuming a monoclinic phase structure with a β angle in the unit cell of 98.65 ± 0.01°, which gradually shifts to higher angle values with increasing pressure. No new peak arises, indicating the structural stability of (4FP)_2_SnI_4_ within the investigated pressure range. Figure 1b presents the monoclinic crystal structure of (4FP)_2_SnI_4_, which was used for the interpretation of the XRD patterns and for DFT simulations presented in this work. Space group of (4FP)_2_SnI_4_ was determined as *P*21/*c* while *a* = 16.654 ± 0.008 Å, *b* = 8.6049 ± 0.0015 Å, *c* = 8.756 ± 0.004 Å, α = 90.00°, γ = 90.00°, and *V* = 1240.3625 Å^3^ Molecular structure of 4FP is presented in Figure S1 in Supporting Information. Figure 1c shows the refined lattice constants *a*, *b*, *c*, and unit cell volume. The lattice parameters of the unit cell were calculated based on the position of reflections (020), (011), (210), (022), and (122̅) and determined for each pressure, which made it possible to plot the change in lattice parameters and unit cell volume as a function of pressure. The derived trends indicate that no phase transition for (4FP)_2_SnI_4_ occurs. Calculated lattice parameters, angles, volume with uncertainties, and R-factor are presented in Table 1 in the Supporting Information. For comparison, theoretical results are also presented in Figure 1c, which are derived from DFT calculations and compare quite well with the experimental trend. Slightly lower values of the lattice parameters from the theory can be explained on the basis of thermal lattice expansion (DFT calculations correspond to 0 K, while the experimental data are obtained at room temperature). Based on the third-order Birch–Murnaghan equation of state, we derived a bulk modulus of 20.09 ± 1.41 GPa, which is comparable to other perovskite structures.^49–51^ Third-order Birch–Murnaghan fitting is presented in Figure S2 in Supporting Information.

**Figure 1:**
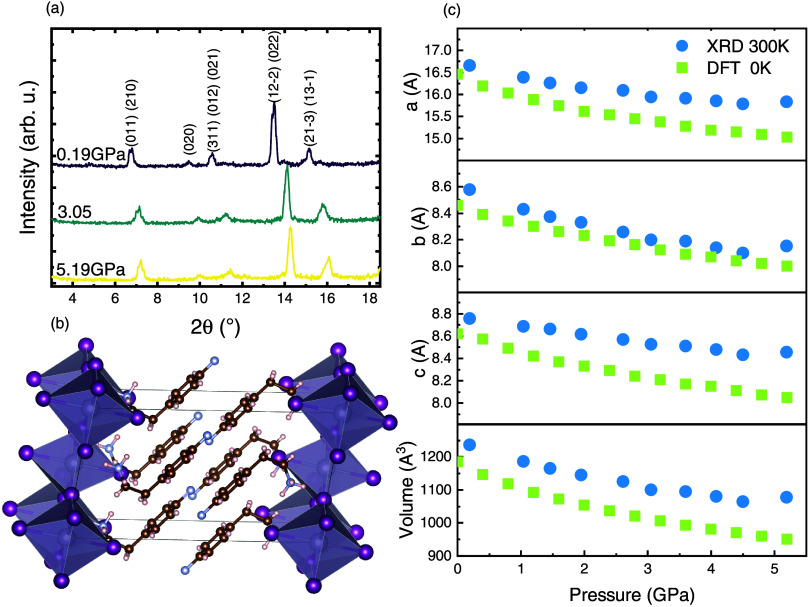
(a) Powder XRD pattern of (4FP)_2_SnI_4_ in three selected pressures, together with interpretation of visible reflections. (b) Crystal structure of (4FP)_2_SnI_4_ used in spectral interpretation and DFT simulations. (c) Lattice parameters and unit cell volume as a function of applied pressure, derived from XRD measurements and DFT calculations.

Figure 2a–d shows normalized PL spectra of (4FP)_2_SnI_4_ as a function of hydrostatic pressure, i.e., isothermal PL measurements at 40, 120, 200, and 300 K, respectively. A discussion about the hydrostaticity of the used pressure medium is presented in Figure S3 in Supporting Information. The temperature dependence of the PL spectrum is shown in Figure S4, where the redshift of the NBE composed of E1 and E2 peaks is observed as the temperature decreases, but its magnitude is negligible compared to the pressure effect studied in this work. In the spectra presented in panels a–d and in Figure S4, E1 and E2 features are marked. This dual peak structure has also been observed in (4FP)_2_SnI_4_ ^12^ other 2D perovskite microcrystals.^52^ Although the specific origins of the E1 and E2 peaks have not been fully clarified, they have been attributed to optical transitions related to isolated Sn–I layers and the edge of the microcrystal, respectively.^12,53^ Depending on the temperature, either E1 or E2 is more intensive and remains visible throughout the studied pressure range. Regardless of this, a huge redshift of the NBE position can be observed as the pressure increases. The pressure-independent peak observed at 1.5 eV is associated with the second order of diffraction of the 405 nm laser used in this measurement. Figure 3a shows the energy position of NBE extracted from the spectra presented in Figure 2a–d. The differences in the spectrum intensities at individual temperatures make it difficult to accurately read the energy position at higher pressures for the 120 K case in Figure 2b. In this case, the NBE signal is overlapped by emission from the ruby sphere (used to measure the pressure) at 1.78 eV. Despite this difficulty, robust red-shift of NBE is evident with increasing hydrostatic pressure. The pressure coefficients for NBE were calculated from the linear fitting of the energy of the NBE versus applied pressure. Obtained values are negative, and they decrease with increasing temperature. At the same time, the intensity of the PL drops, whereas peak width increases with applied pressure.

**Figure 2:**
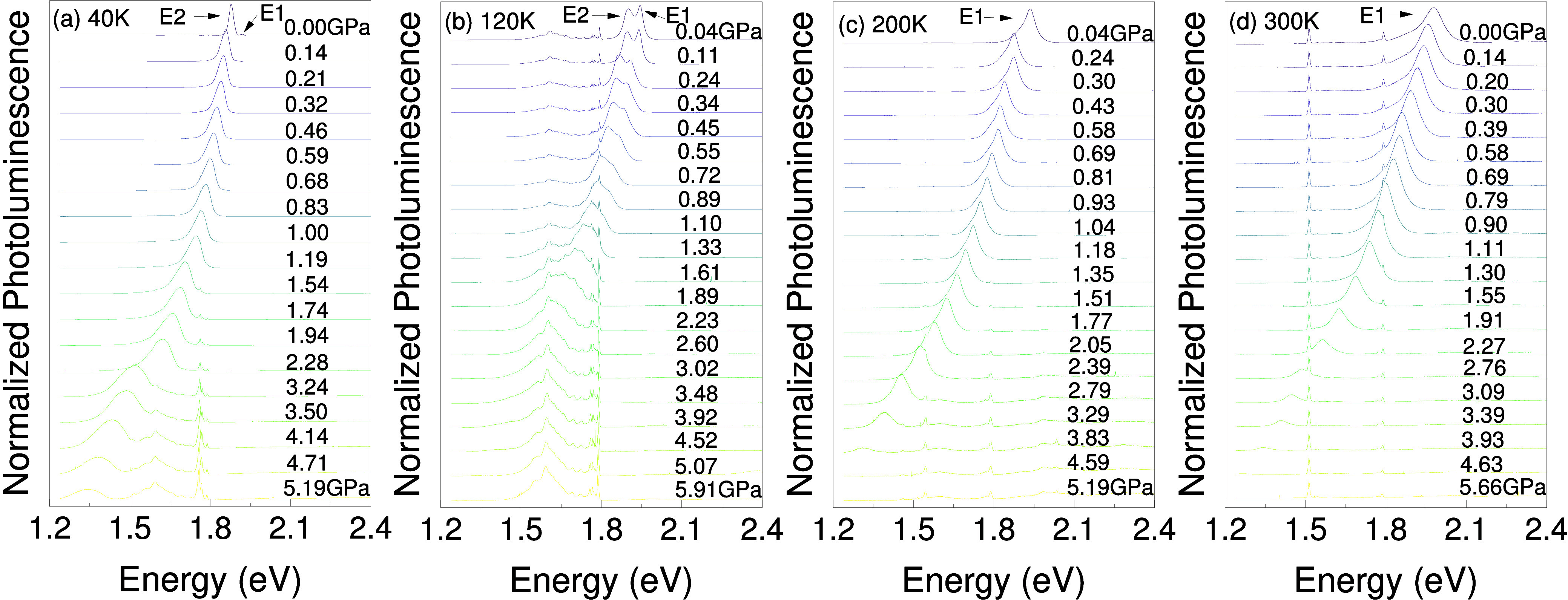
Photoluminescence spectra of (4FP)_2_SnI_4_ as a function of applied pressure at four different temperatures: 40, 120, 200, and 300 K (panels a–d respectively).

**Figure 3:**
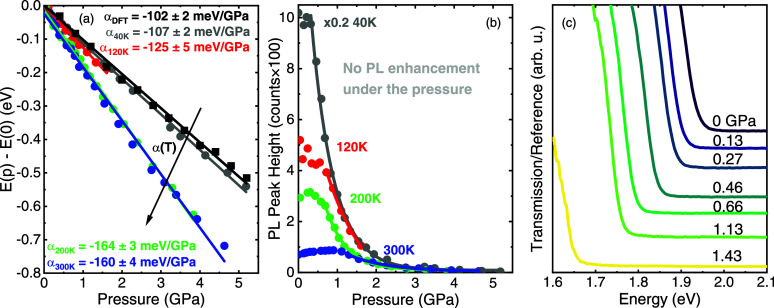
(a) Band gap change Δ*E* = *E*(*P*) – *E*(*P* = 0) as a function of pressure derived from PL spectra for different temperatures (circles), together with linear fits (lines). Clear dependence of the pressure coefficient on temperature can be observed. Results of DFT simulation are also presented as black squares. (b) PL peak high for different temperatures and pressures. (c) Transmission spectra near the absorption edge for different values of applied pressure.

Figure 3b presents the temperature dependence of the intensity of the NBE at varying hydrostatic pressure. As can be seen, the NBE intensity drops rapidly with increasing pressure, and at values around 3 GPa, it becomes difficult to estimate with good accuracy. For each temperature, this trend is maintained. In contrast to many other halide perovskites, no pressure-induced PL enhancement phenomenon or a drastic increase in the band gap value is observed,^54,55^ at least up to around 5 GPa of applied pressure.

The very strong red-shift of NBE is attributed to the band gap narrowing in (4FP)_2_SnI_4_ under hydrostatic pressure. In order to further study the pressure-induced changes in the band gap, transmission spectra under hydrostatic pressure were measured. Figure 3c shows normalized transmission spectra for (4FP)_2_SnI_4_ as a function of hydrostatic pressure taken at 300 K. It can be observed that as the pressure increases, the band gap of (4FP)_2_SnI_4_ red-shifts, similar to the behavior of NBE in PL measurements. The pressure coefficient extracted from transmission measurements is equal to −184 ± 16 meV/GPa, which is a value similar to that extracted from room-temperature PL spectra (−160 ± 4 meV/GPa). This indicates a strong correlation of the NBE and the absorption edge. The method by which absorption edge was determined is shown in Figure S5. In Figure S6 optical micrographs of the (4FP)_2_SnI_4_ sample are presented. The change in the crystal color from red at 0 GPa to black at 1.43 GPa is in line with the band gap narrowing observed in transmission and PL.

To obtain further validation of our experimental results, we performed DFT modeling of the band structure evolution under pressure. Figures 4a,b show representative band structures for 0 and 4.8 GPa of applied pressure, respectively. For the studied pressure range of 0–5.2 GPa, the fundamental band gap is direct and decreases with applied pressure, which is presented in Figure 3a. Derived from calculations, the pressure coefficient is in excellent agreement with low-temperature experimental results, despite the simple level of DFT theory used in this work (see SI for details).

**Figure 4:**
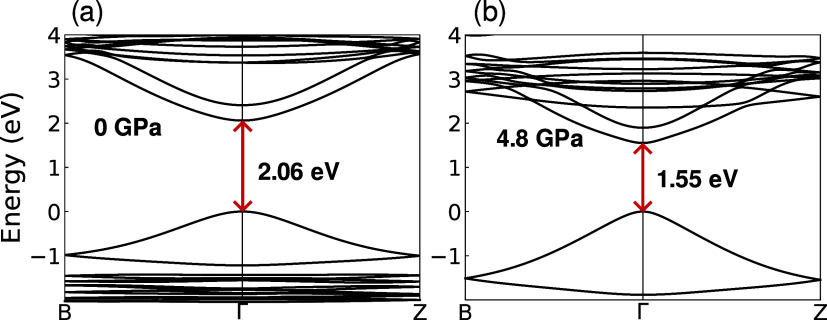
Band structure of (4FP)_2_SnI_4_ for (a) 0 and (b) 4.8 GPa applied pressure. The band gap remains direct for whole range of studied pressures and exhibit significant redshift.

It is worth empathizing that the pressure coefficient derived from measurements and theoretical modeling not only has great absolute value but also stays constant for a wide range of pressures, which gives an opportunity for large band gap tuning by pressure in a linear fashion. Considering the properties of (4FP)_2_SnI_4_ compared to other halide perovskites, it is clear that the value of the pressure coefficient and band gap tunability are among the highest reported to date, making this material a promising candidate for pressure-sensitive applications. The constant pressure coefficient in such a wide range of pressures is also unique among this material group. As mentioned in the Abstract, we attribute this, on the one hand, to its 2D structure and, on the other hand, to the tin occupying metal sites.

It is well-known that the band edges near the fundamental gap in 2D halide perovskites are made from orbitals of atoms from inorganic layers. In the case of (4FP)_2_SnI_4_, the valence band maximum is a combination of *s* orbitals of tin and *p* orbitals of iodine, while the conductive band minimum is mainly from *p* orbitals of tin. Therefore, in order to understand the evolution of the band gap under pressure, it is crucial to examine the changes in the bonds between iodine and tin. Our DFT calculations show that the lengths of these bonds change by almost 0.2 Å, while the changes in the Sn–I bond angles are smaller than 2° in the pressure range studied. Another observation is that the Sn–I bonds directed to the organic spacer exhibit approximately two times smaller changes in lengths (∼0.1 Å), which can be explained by noting that in the direction perpendicular to the inorganic layer, most of the pressure is used to squeeze the organic spacer. This picture is in agreement with the mechanism proposed in previous work^51^ in which authors show that the initial band gap dependence of perovkites is a steady redshift, accompanied by metal-halide bond shortening (at least if the material does not undergo phase transition in this initial pressure range). According to previous studies^51^ at some pressure another regime occurs, after which the band gap increases with pressure due to the change in angles between Sn–I bonds. In our study, (4FP)_2_SnI_4_ remains in the first region up to 5 GPa, which stems from the large width of the organic spacer relative to the width of the inorganic part and the presence of tin instead of lead, as smaller atoms give more space for organic cations to fit in. Such conditions provide material with a linear pressure coefficient and great band gap tunability in a wide pressure range, in agreement with chemical trends derived in previous studies.^51^

In conclusion, a comprehensive study of (4FP)_2_SnI_4_ optical properties under hydrostatic pressure has been carried out. PL spectra show that the NBE position redshifts dramatically under the influence of pressure. The obtained pressure coefficient is negative and increases with increasing temperature. Transmission measurement shows that the near band emission is related to the band gap, and for relatively small value of increased pressure we observe strong redshift resulting in color change. Finally, we validated our observation by DFT simulations and explained a possible reason for such behavior based on geometrical arguments. The pressure at which irreversible amorphization of the crystal occurs has not been determined, and no phase transitions have been observed up to 5 GPa. Compared to other halide perovskites, (4FP)_2_SnI_4_ possesses one of the highest band gap tunabilities reported to date, which marks it as a promising candidate for pressure-sensor applications.

## Supplementary Material



## Notes

Detailed materials and methods; experimental layout; additional photoluminescence spectra of (4FP)_2_SnI_4_; 4FP molecular structure; determination of the absorption edge; optical micrographs of (4FP)_2_SnI_4_ sample mounted in the DAC; analysis of ruby fluorescence peaks; comment about hydrostaticity of Daphne 7575; third-order Birch–Murnaghan fitting; calculated lattice parameters, angles, and volume with uncertainties as a function of pressure (PDF)
