# Supplementary material for: Giant Band Gap Narrowing under Hydrostatic Pressure in (4FP)2SnI4 Halide Perovskite
Source: J Phys Chem Lett. 2025 Jun 16;16(25):6372–7. doi: 10.1021/acs.jpclett.5c00903 (PMC12207673; doi:10.1021/acs.jpclett.5c00903)
Supplement: Supplementary file 1 [file jz5c00903_si_001.pdf]

Supporting Information for

Giant band gap narrowing under hydrostatic pressure in $(4FP)_2SnI_4$ halide perovskite

*Rafał Bartoszewicz,[1, *] Jakub Ziembicki,[1] Ewelina Zdanowicz,[1] Artur P. Herman,[1] Jarosław Serafińczuk,[2] Jesús Sánchez-Diaz,[3] Samrat Das Adhikari,[3,4] Iván Mora-Seró,[3] and Robert Kudrawiec[1, **]*

[1]Department of Semiconductor Materials Engineering, Wroclaw University of Science and Technology, Wybrzeże Wyspiańskiego 27, 50-370 Wrocław, Poland

[2]Department of Nanometrology, Wroclaw University of Science and Technology, Janiszewskiego 11/17, 50-372 Wrocław, Poland

[3]Institute of Advanced Materials (INAM), Universitat Jaume I. Av. De Vicent Sos Baynat, Castellón de la Plana, 12006 Spain

[4]Institute of Physical Chemistry, Polish Academy of Sciences, Warsaw 01-224, Poland

* e-mail address: rafal.bartoszewicz@pwr.edu.pl

** e-mail address: robert.kudrawiec@pwr.edu.pl

KEYWORDS: Perovskites, Photoluminescence, Absorption, XRD, Hydrostatic pressure

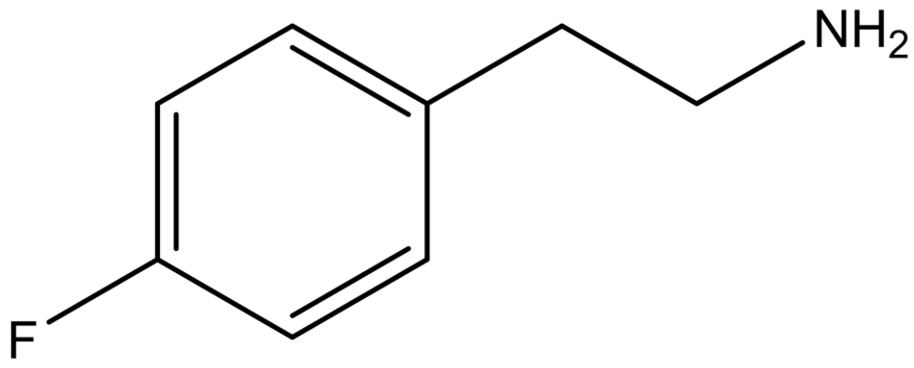

Figure S1. 4FP molecular structure

Table 1. Calculated lattice parameters, angles, volume and R-factor of $(4FP)_2SnI_4$ as a function of applied pressure

Pressure [GPa]	a [Å]	Δa [Å]	b [Å]	Δb [Å]	c [Å]	Δc [Å]	β [°]	Δβ [°]	c/a	a/b	c/b	V (Å³)	ΔV (Å³)	R-factor [%]
0.19	16.654	0.008	8.5774	0.0015	8.756	0.004	98.65	0.01	0.5257	1.9415	1.0207	1236.41	0.85	0.9
1.04	16.388	0.008	8.4298	0.0015	8.685	0.004	98.72	0.01	0.5299	1.9440	1.0302	1185.85	0.82	0.68
1.46	16.255	0.008	8.3722	0.0014	8.663	0.004	98.80	0.01	0.5329	1.9415	1.0346	1164.96	0.81	0.64
1.95	16.151	0.008	8.3294	0.0014	8.614	0.004	98.84	0.01	0.5333	1.9390	1.0341	1144.96	0.79	0.62
2.61	16.087	0.008	8.2585	0.0014	8.569	0.004	98.91	0.01	0.5326	1.9479	1.0375	1124.60	0.78	0.6
3.05	15.939	0.008	8.1977	0.0014	8.524	0.004	98.95	0.01	0.5347	1.9443	1.0397	1100.11	0.76	0.59
3.60	15.909	0.008	8.1868	0.0014	8.509	0.004	98.98	0.01	0.5348	1.9432	1.0392	1094.57	0.76	0.58
4.08	15.856	0.008	8.1397	0.0014	8.479	0.004	99.00	0.01	0.5347	1.9480	1.0416	1080.76	0.75	0.57
4.50	15.777	0.008	8.0991	0.0014	8.432	0.004	99.03	0.01	0.5344	1.9480	1.0410	1063.99	0.74	0.56
5.19	15.827	0.008	8.1517	0.0014	8.454	0.004	98.99	0.01	0.5341	1.9415	1.0370	1088.21	0.75	0.56

Calculations were performed considering 5 lattice planes: (011), (210), (022), (020) and (12-2), which allowed us to accurately determine both the lattice parameters and the beta angle. In addition, measurement uncertainties were calculated for the measurement step used of 0.0167 degrees.

The R-factor is defined as:

$$R = \frac{\sum |d_{observed} - d_{calculated}|}{\sum d_{observed}} \cdot 100\%$$

Where $d_{observed}$ is derived from the measured 2θ angles using Bragg's law, and $d_{calculated}$ is computed from the refined lattice parameters.

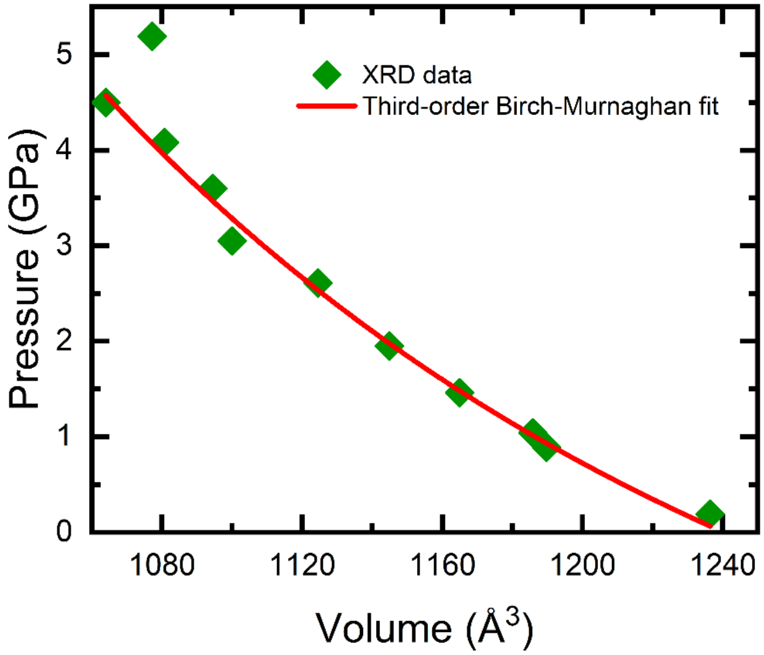

Figure S2. Third-order Birch-Murnaghan fitting of XRD data.

To determine bulk modulus value of $(4FP)_2SnI_4$ we used third-order Birch-Murnaghan EoS :

$$P(V) = \frac{3}{2}B_0\left[\left(\frac{V_0}{V}\right)^{7/3} - \left(\frac{V_0}{V}\right)^{5/3}\right] \times \left\{1 + \frac{3}{4}(B_0' - 4)\left[\left(\frac{V_0}{V}\right)^{2/3} - 1\right]\right\}$$

The approximation was calculated using Origin and also python software. After refitting the third-order Birch-Murnaghan function with a fixed value of V_0 = 1240.3625 Å^3 we received :

B_0 = 20.09 ± 1.41 GPa

$B_0{}'$ = 5.14 ± 1.12

The measurement at 5.19 GPa was excluded after calculations due to a likely error in determining the pressure within the DAC.

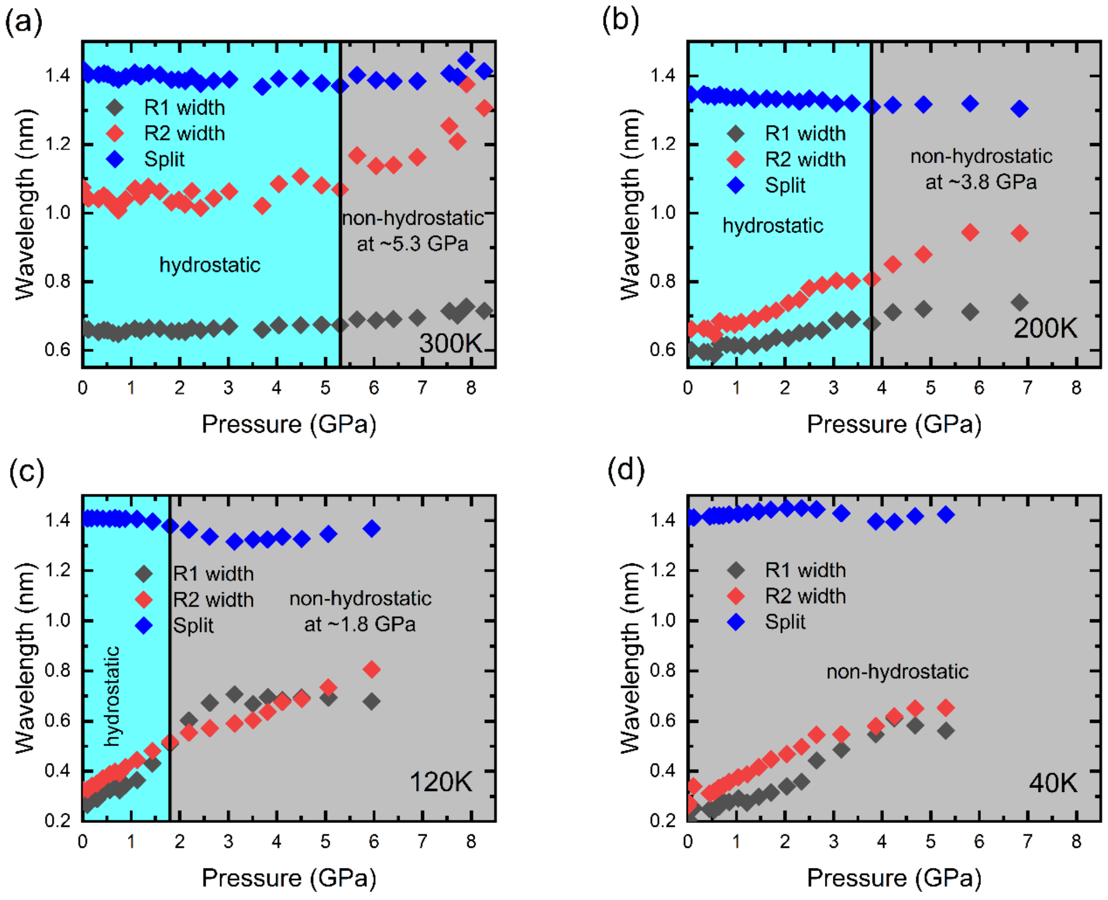

Figure S3. Analysis of ruby R1 and R2 peaks split and broadening at (a) 300, (b) 200, (c) 120, and (d) 40 K.

Figure S3 presents the analysis of the applied pressure using ruby fluorescence. Daphne 7575 is not strictly hydrostatic at low temperatures, and its ability to maintain hydrostatic conditions decreases significantly with both temperature reduction and pressure increase [1,2]. However, while Daphne 7575 does not remain fully hydrostatic at high pressures and low temperature, it maintains quasi-hydrostatic conditions, owing to its relatively low shear strength and soft solidification behavior [2,3]. From R1 and R2 peaks split and broadening we determined solidification points of Daphne 7575 at (a) ~5.3, (b) ~3.8, (c) ~1.8 and (d) 0 GPa for 300, 200, 120 and 40 K respectively. The pressure coefficients we investigated are not highly sensitive to moderate non-hydrostatic stress. As far as we know, such conditions should lead to nonlinear band gap shifts, splitting of emission lines, amorphization, or appearing defect emission due to uneven stress, which could be reported as the presence of phase transition in this material [4-7]. Such effects were not observed in our measurements. Our results remain consistent with theoretical expectations, which support their validity even in the presence of quasi-hydrostatic conditions. Moreover, for perovskite materials such medium is still second best choice (first choice is helium) as it not influence the condition of measured sample, as it is in the case of 4:1 methanol-ethanol mixture [8].

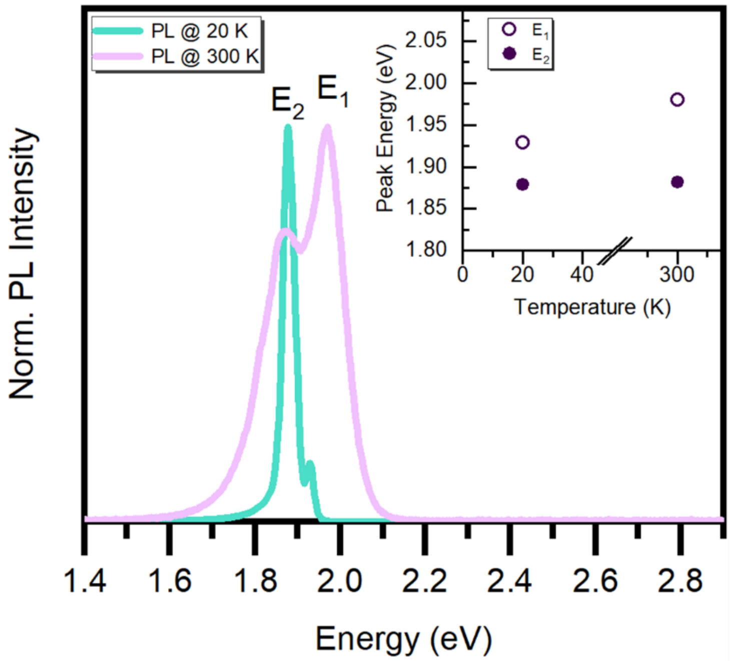

Figure S4. Normalized photoluminescence spectra of $(4FP)_2$ SnI_4. The inset shows energies of E_1 and E_2 at investigated temperatures.

Figure S4 compares the PL spectra recorded at 20 and 300 K, revealing two distinct peaks, E_1 and E_2, at both temperatures. The inset illustrates the temperature dependence of the extracted energies for E_1 and E_2. Notably, E_1 exhibits a blueshift with increasing temperature, while E_2 remains nearly constant. The PL spectra in Figure S1 indicate that $(4FP)_2SnI_4$ maintains strong emission properties across a wide temperature range.

Figure S5 illustrates the method used to determine the energy shift of the absorption edge. Using a linear regression fit, we estimated the energy value from the square of the absorption coefficient (α^2), where α is defined according to the formula:

$$\alpha = -\frac{1}{d} ln \frac{T}{(1-R)^2}$$

where d is the sample thickness (here 50μm), T means– transmission and, R – reflectance signal.

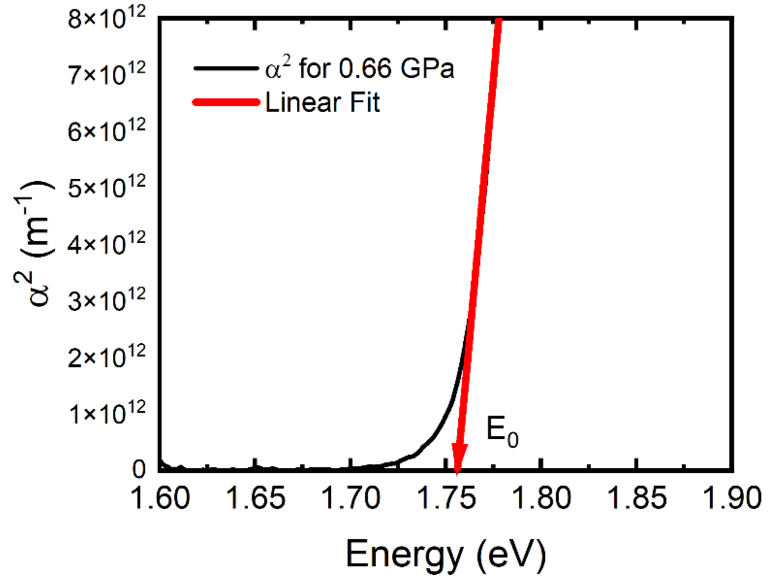

Figure S5. Determination of the absorption edge at 0.66 GPa.

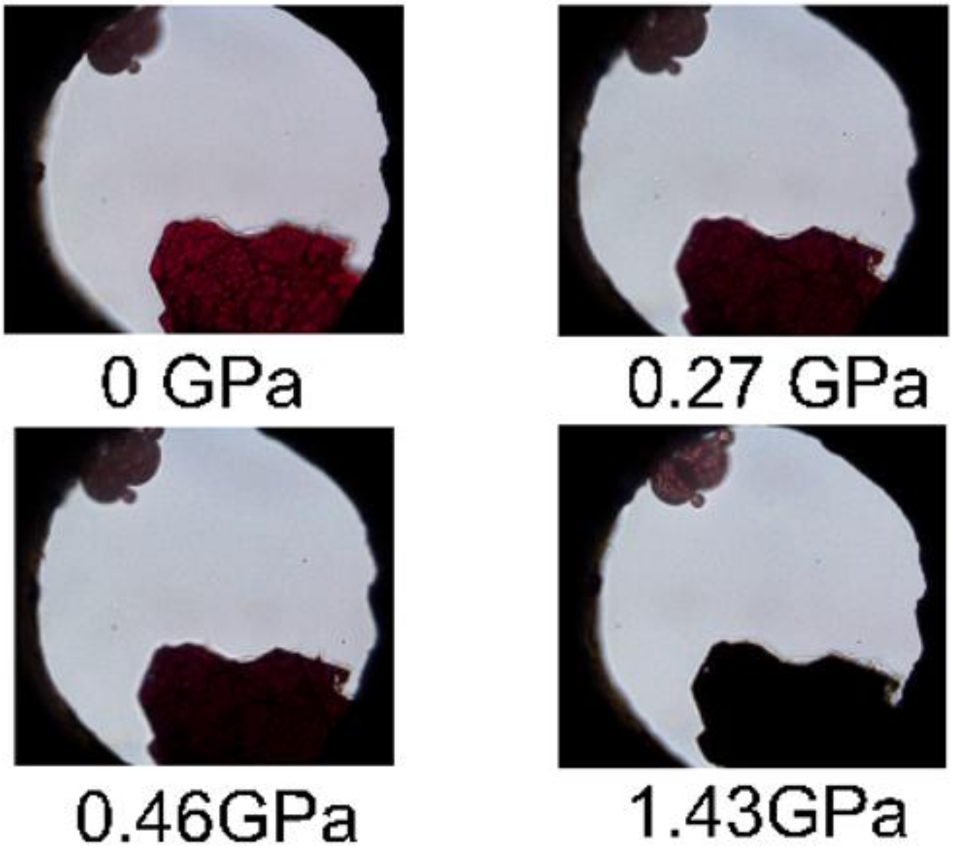

Figure S6. View on the sample for different values of pressure.

MATERIALS AND METHODS

Experimental details:

Materials: Acetic acid (AcOH, 99-100 %, sigma aldrich), hydroiodic acid (HI; 57 wt. % in H_2O, distilled, stabilized, 99.95 %), hypophosphorus acid (H_3PO_2; 50wt. % in H_2O), Tin (II) oxide (SnO; 99%, Alfa Aesar), 4-fluorophenethylamine (4FPEA; TCI) was purchased from. All materials were use as received with no further purifications.

Synthesis of $(4FP)_2SnI_4$ microcrystals powder:

Synthesis of $(4FP)_2SnI_4$ was carried out following the literature report [8, 9]. A mixture consisting of 3 mL of acetic acid, 0.2 mL of hydroiodic acid (HI), and 0.05 mL of hypophosphorous acid (H_3PO_2) was combined in a three-necked flask under an inert nitrogen (N_2) atmosphere. The mixture was stirred at room temperature for 30 minutes, during which a transition from a faint red to a colorless appearance indicated the stabilization of HI in the presence of H_3PO_2. Next, 134 mg of tin(II) oxide (SnO) was introduced into the flask, and the temperature was gradually increased to 100 °C. The solution was stirred for an additional 30 minutes until it developed an orange color. Subsequently, 0.26 mL of 4-fluorophenylethylamine (4FPEA) was added, and the reaction temperature was further raised to 135 °C. The mixture was maintained under these conditions for 15 minutes. Upon completion of the reaction, the solution was rapidly cooled using an ice bath for approximately 2 minutes. The resulting product was collected via vacuum suction filtration. To eleiminate any excess iodine, the collected powder was washed three times with hexane, followed by additional vacuum suction filtration. Finally, the purified product was dried.

Temperature and pressure dependent PL

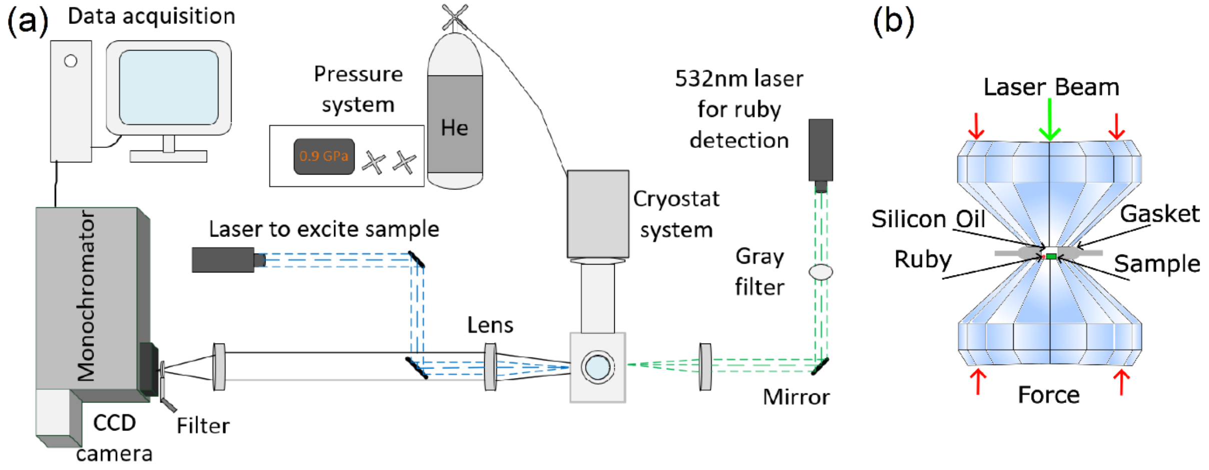

Figure S7. (a) The scheme of the experimental setup for hydrostatic pressure-dependent photoluminescence (b) Cross-section of a diamond anvil cell.

Figure S7 (a) presents schematically the experimental setup of temperature- and pressure-dependent PL carried out in this work. Figure S7 (b) shows a cross-section of a diamond anvil cell (DAC). PL measurements were performed in Diacell design DAC by ‘Almax easyLab’. DAC used in this work is made of beryllium copper (BeCu) alloy, which allows to perform measurements at cryogenic temperature (here 40 K). Diamond with 650 μm culet size was used. The pressurizing mechanism in the DAC was driven using a gas membrane. The pressure in DAC was controlled by the amount of helium injected into the gas membrane. On top of the bottom diamond culet, a gasket was placed. It is a thin sheet (circa 50 μm thickness) of inconel material with a central hole of about 1/3 culet size, where sample, ruby sphere, and silicon oil were placed. Gaskets were prepared by pressing diamonds into a thin sheet, followed by creating a central hole using mechanical drilling. Daphne 7575 was used as the pressure-transmitting medium, which solidifies at about 5.3GPa at room temperature. Whole setup was cooled down in a cryostat with a closed-loop liquid helium system. For PL experiments, the 405 nm CW (with power of 100 μW) laser was used to excite the studied material. Emitted light was dispersed through a 0.5 m Andor monochromator with a 150 l/mm diffraction grating blazed at 500 nm. The signal was recorded by a Si CCD camera cooled to -70 °C by Peltier module. The luminescence of the ruby R1 line at ~1.78 eV (at room temperature) was excited by the 532 nm CW laser, dispersed on the 600 l/mm diffraction grating blazed at 500 nm and detected by CCD camera. To determine pressure value inside DAC we detect pressure-induced redshift of ruby sphere R1 line with calibration taken from Shen [10]. The temperature shift correction of the R1 ruby line was taken from Datchi [11].

Pressure dependent transmission

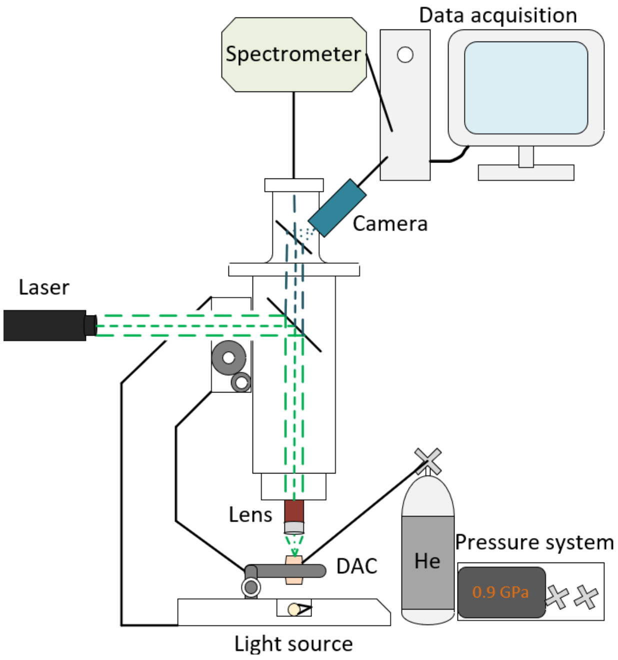

Figure S8. Scheme of the system used for transmission measurements under hydrostatic pressure.

Figure S8 presents a scheme of the experimental setup for hydrostatic pressure-dependent transmission at room temperature. The optical microscope NADE NMM-800TRF and Mitutoyo M PLAN APO NIR 50x/0.42 f = 200 lens was used to properly focus on sample placed inside the DAC chamber. Transmission measurements were carried out using the previously mentioned DAC which was also used for the PL measurements, with Daphne 7575 serving as the pressure-transmitting medium. For transmission measurements, the halogen lamp (with voltage of 24 V and power 100 W) was used as a light source. Transmitted light was recorded by a StellarNet Blue-Wave UVIS 200 spectrometer with a 600 l/mm grating and 200 nm slit with resolution of 1.0. Delta Optical DLT-CAM PRO 3 MP USB 3.0 camera allowed us to investigate the color change of the perovskite. To excite the R1 luminescence of the ruby, a 532 nm CW laser was used (emission power 1 mW). In this case, AVANTES ULS4096CL-RS-EVO with a DCL-UV/VIS-200 600 l/mm diffraction grating blazed at 300 nm was used as a detector. The investigated spectral range is determined by the upper optical limit of the Mitutoyo NIR lens used and the cutoff energy of the optical microscope (approximately 1.6 eV).

Theoretical calculations

DFT calculations have been done in VASP code, using PAW method [12-15]. For geometrical optimization we used PBE with D3 van der Waals correction [16-17], with 600 eV cutoff energy, 3x3x6 kpoint grid, 10^{-7} eV energy tolerance for SCF and 10^{-2} eV/A force tolerance for geometry optimization. Then band structures have been calculated with PBE functional, using the same numerical parameters. To correct the band gap, we used scissor shift to set the band gap to the value derived on the PBE0 level [18] on the same geometry. The spin-orbit coupling (SOC) is included in band structure calculations, as it is well known that SOC greatly affect the band structure in tin and lead based perovskites.

Pressure dependent X-ray diffraction

Structural studies with X-ray diffraction, were carried out using an Malvern-Panalytical Empyrean diffractometer. A molybdenum x-ray tube with $Mo_{K\alpha 1}$ = 0.7093197 Å was used in the study. Pressure was generated by an 'Almax easyLab' Bragg-Mini DAC with diamond culet size of 600 µm allowing pressures up to 20 GPa. Daphne 7575 was used as the pressure-transmitting medium, which solidifies at about 5.3 GPa at room temperature. An x-ray tube was set to point focus and a 135 mm long mono-capillary collimator with an exit beam diameter of 0.3 mm were used to form the incident beam. The diffraction curves were recorded by a multi-strip detector 1Der. Measurements were carried out at range from 3 to 19 degrees which allowed all the strongest reflections for the investigated material to be observed. Counting time was 3400 s/point.

References:

[1] Staško, D., Prchal, J., Klicpera, M., Aoki, S., & Murata, K. (2020). Pressure media for high pressure experiments, Daphne Oil 7000 series. *High Pressure Research*, *40*(4), 525–536. https://doi.org/10.1080/08957959.2020.1825706

[2] Naoyuki Tateiwa and Yoshinori Haga 2010 J. Phys.: Conf. Ser. 215 012178

[3] Y Nakamura et al 2010 J. Phys.: Conf. Ser. 215 012176

[4] Tuson Park et al 2008 J. Phys.: Condens. Matter **20** 322204

[5] Yu, W, Aczel, A. A, Williams, T. J, Bud'ko, S. L, Ni, N, Canfield, P. C, Luke and G. M. Phys. Rev. B 79 020511(R) (2009)

[6] J. Gong, H. Zhong, C. Gao, J. Peng, X. Liu, Q. Lin, G. Fang, S. Yuan, Z. Zhang, X. Xiao, Pressure-Induced Indirect-Direct Bandgap Transition of $CsPbBr_3$ Single Crystal and Its Effect on Photoluminescence Quantum Yield. *Adv. Sci.* 2022, 9, 2201554. https://doi.org/10.1002/advs.202201554

[7] Xujie Lu et al 2017 Chem. Sci. 8, 6764-6776

[8] J. Sanchez-Diaz, J. Rodriguez-Pereira, S. Das Adhikari, I. Mora-Seró, Synthesis of Hybrid Tin-Based Perovskite Microcrystals for LED Applications. Adv. Sci. 2024, 11, 2403835. https://doi.org/10.1002/advs.202403835

[9] Y. Su, J. Yang, G. Liu, W. Sheng, J. Zhang, Y. Zhong, L. Tan, Y. Chen, Acetic Acid-Assisted Synergistic Modulation of Crystallization Kinetics and Inhibition of Sn^{2+} Oxidation in Tin-Based Perovskite Solar Cells, Adv. Funct. Mater. 2022, 32, 2109631. https://doi.org/10.1002/adfm.202109631

[10] Shen, G.; Wang, Y.; Dewaele, A.; Wu, C.; Dayne, E.; Eggert, J.; Klotz, S.; Dziubek, K. F.; Loubeyre, P.; Fat, V.; Asimow, P. D.; Mashimo, T.; Wentzcovitch, R. M. M.; Toward an International Practical Pressure Scale: A Proporsal for an IPPS Ruby Gauge (IPPS-Ruby2020). High Press. Res. 2020, 40, 299-314 10.1080/08957959.2020.1791107

[11] Datchi, F.; Dewaele, A.; Loubeyre, P.; Letoullec, R.; Godec, Y. Le.; Canny, B.; Optical Pressure Sensors for High-Pressure-High-Temperature Studies in a Diamond Anvil Cell. High Press. Res. 2007, 27, 447-463.c https://doi.org/10.1080/08957950701659593

[12] G. Kresse and J. Hafner, Ab initio molecular dynamics for liquid metals, Phys. Rev. B 47, 558 (1993).

[13] G. Kresse and J. Furthm¨uller, Efficient iterative schemes for ab initio total-energy calculations using a plane-wave basis set, Phys. Rev. B 54, 11169–11186 (1996).

[14] G. Kresse and J. Furthm¨uller, Efficiency of ab-initio total energy calculations for metals and semiconductors using a plane-wave basis set, Computational Materials Science 6, 15 (1996).

[15] G. Kresse and D. Joubert, From ultrasoft pseudopotentials to the projector augmented-wave method, Phys. Rev. B 59, 1758 (1999).

[16] J. P. Perdew, K. Burke, and M. Ernzerhof, Phys. Rev. Lett., **77**, 3865 (1996).

[17] Grimme, S., Ehrlich, S. and Goerigk, L. (2011), Effect of the damping function in dispersion corrected density functional theory. J. Comput. Chem., 32: 1456-1465.

[18] C. Adamo and V. Barone, Phys. Rev. Lett., **110**, 6158 (1999).
